# Supplementary material for: Self-assembly of CIP4 drives actin-mediated asymmetric pit-closing in clathrin-mediated endocytosis
Source: Nat Commun. 2023 Aug 1;14:4602. doi: 10.1038/s41467-023-40390-y (PMC10393992; doi:10.1038/s41467-023-40390-y)
Supplement: Supplementary file 1 — Supplementary Information [file 41467_2023_40390_MOESM1_ESM.pdf]

Supplementary Information for

Self-assembly of CIP4 drives actin-mediated asymmetric pit-closing in clathrin-mediated endocytosis

Yiming Yu and Shige H. Yoshimura*

Graduate School of Biostudies, Kyoto University, Kyoto 606-8501, Japan

*Corresponding author

Shige H. Yoshimura

Graduate School of Biostudies, Kyoto University, Kyoto 606-8501, Japan

E-mail: yoshimura@lif.kyoto-u.ac.jp

Tel & Fax: +81-75-753-7906

ORCID: 0000-0001-6033-1301

This PDF file includes:

Supplementary Figures 1 to 6 and Supplementary Table 1

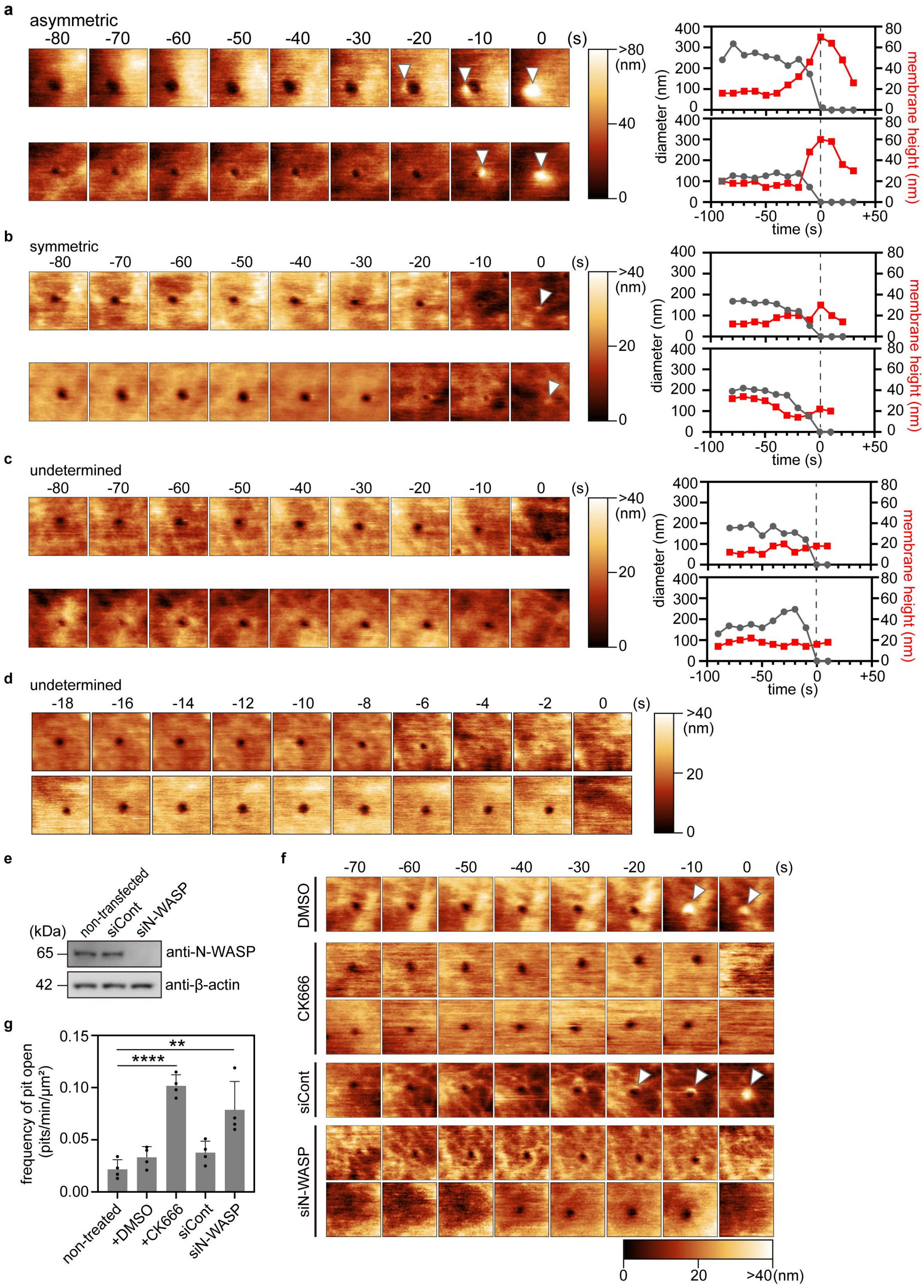

a
asymmetric
-80 -70 -60 -50 -40 -30 -20 -10 0 (s)
>80 (nm)
40
0
diameter (nm)
membrane height (nm)
time (s)
b
symmetric
-80 -70 -60 -50 -40 -30 -20 -10 0 (s)
>40 (nm)
20
0
c
undetermined
-80 -70 -60 -50 -40 -30 -20 -10 0 (s)
d
undetermined
-18 -16 -14 -12 -10 -8 -6 -4 -2 0 (s)
e
(kDa)
non-transfected
siCont
siN-WASP
65
anti-N-WASP
42
anti-β-actin
f
-70 -60 -50 -40 -30 -20 -10 0 (s)
DMSO
CK666
siCont
siN-WASP
0 20 >40(nm)
g
frequency of pit open (pits/min/μm²)
****
**
non-treated
+DMSO
+CK666
siCont
siN-WASP

Supplementary Figure. 1 Asymmetric closing pattern of the CCP.

a–c, Additional examples of the time-lapse HS-AFM images of asymmetric (a), symmetric (b), and undetermined (c) closing patterns obtained from Cos7 cells. Details of the experimental conditions and data presentations are the same as those in Figs. 1a, 1c.

d, Time-lapse HA-AFM images of CCPs with the undetermined closing pattern at a scanning rate of 2 s/frame. Time 0 was when the pit completely closed on the HS-AFM image. The height information of the AFM image is presented using a colour bar. The image size was 1.0×1.0 μm^2.

e, Knockdown efficiency of N-WASP. The total cell lysate of non-transfected Cos7 cells and Cos7 cells transfected with siRNA for the control (luciferase) or N-WASP was prepared 48 h after the transfection and then subjected to the western blot analysis using an anti-N-WASP antibody. β-actin was used as an internal control. The experiment was repeated three times and similar results were obtained.

f, Time-lapse HS-AFM images obtained from Cos7 cells treated with DMSO or CK666 and cells transfected with siRNA for the control (luciferase) or N-WASP. Images were taken every 10 s. Time 0 was when the pit completely closed on the HS-AFM image. Arrowheads indicate the membrane bulge, and the height information of the AFM image is presented using a colour bar. Image size: 1.0×1.0 μm^2.

g, The frequency of CCP formation in non-treated Cos7 cells, the cells treated with DMSO or CK666, and the cells transfected with siRNA for the control (luciferase) or N-WASP were quantified and summarised. Data are presented as mean ± standard deviation from four independent experiments. All data points are shown. *P* values were calculated using the two-tailed Student's *t*-test, **: $P < 0.01$; ****: $P < 0.0001$, $\alpha = 0.05$. The exact *P* values are provided in Source Data.

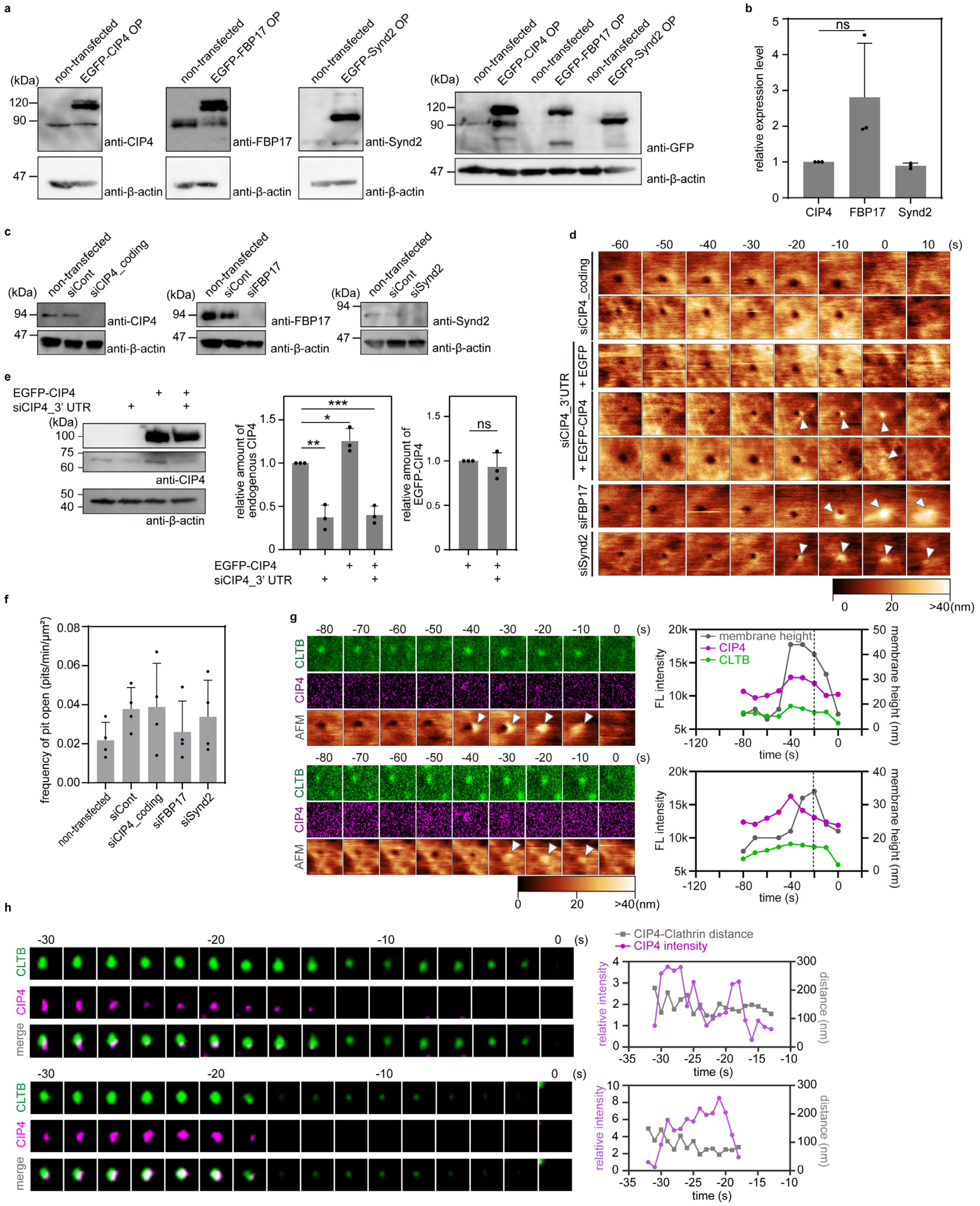
a
non-transfected
EGFP-CIP4 OP
EGFP-FBP17 OP
EGFP-Synd2 OP
(kDa)
120
90
47
anti-CIP4
anti-FBP17
anti-Synd2
anti-GFP
anti-β-actin
b
ns
relative expression level
CIP4
FBP17
Synd2
c
siCont
siCIP4_coding
siFBP17
siSynd2
94
anti-CIP4
anti-FBP17
anti-Synd2
anti-β-actin
d
-60
-50
-40
-30
-20
-10
0
10
(s)
siCIP4_coding
siCIP4_3'UTR
+ EGFP
+ EGFP-CIP4
siFBP17
siSynd2
0
20
>40(nm)
e
EGFP-CIP4
siCIP4_3' UTR
(kDa)
100
75
60
50
40
anti-CIP4
anti-β-actin
relative amount of endogenous CIP4
relative amount of EGFP-CIP4
**
*
***
ns
f
frequency of pit open (pits/min/μm²)
non-transfected
siCont
siCIP4_coding
siFBP17
siSynd2
g
-80
-70
-60
-50
-40
-30
-20
-10
0
(s)
CLTB
CIP4
AFM
membrane height
FL intensity
membrane height (nm)
time (s)
h
-30
-20
-10
0
(s)
CLTB
CIP4
merge
CIP4-Clathrin distance
CIP4 intensity
relative intensity
distance (nm)
time (s)

Supplementary Figure. 2 CIP4 is necessary for the asymmetric bulge.

a, The endogenous amount of CIP4, FBP17, and Synd2 in Cos7 cells. The total cell lysate of Cos7 cells expressing EGFP-fused CIP4, FBP17, or Synd2 was subjected to western blotting using anti-CIP4, -FBP17, or -Synd2 antibodies, respectively. The same lysate was also blotted with an anti-GFP antibody for reference. The amount of β-actin was detected with an anti-β-actin antibody as an internal control.

b, The endogenous amount of CIP4, FBP17, and Synd2 obtained from western blotting is indicated in (a). Data from the three independent experiments are summarised. *P* values were calculated using the two-tailed Student's *t*-test, ns: not statistically significant, $\alpha = 0.05$. The exact *P* values are provided in Source Data. Data are presented as mean ± standard deviation from three independent experiments as a relative value to that of CIP4. All data points are shown.

c, Knockdown efficiency of CIP4, FBP17, and Synd2 in Cos7 cells. Total cell lysate of non-transfected Cos7 cells and Cos7 cells transfected with siRNA for the control (luciferase), CIP4, FBP17, or Synd2 was prepared 48 h after the transfection and then subjected to the western blot analysis using anti-CIP4, FBP17, or Synd2 antibodies. β-actin was detected as an internal control. The experiment was repeated once.

d, Additional examples of the time-lapse HS-AFM images obtained from Cos7 cells transfected with siRNA against CIP4 (siCIP4_coding), Synd2, or FBP17 and Cos7 cells transfected with siRNA targeting the 3′UTR sequence of CIP4 (siCIP4_3′UTR), followed by the overexpression of EGFP or EGFP-fused CIP4. Details of the experimental conditions and data presentations are the same as those in Fig. 2c.

e. Knockdown efficiency of CIP4 using siRNA targeting the 3′UTR sequence of CIP4 (siCIP4_3′UTR). Total cell lysate of non-transfected Cos7 cells, cells transfected with siCIP4_3′UTR, cells transfected with siCIP4_3′UTR followed by the transfection of plasmids encoding EGFP or EGFP-fused CIP4 was prepared 48 h after the transfection and then subjected to the western blot analysis using anti-CIP4 antibodies. β-actin was used as an internal control. The relative amount of endogenous CIP4 and overexpressed EGFP-CIP4 is summarised in the right panels. Data from the three independent experiments are summarised and presented as mean ± standard deviation. All data points are shown. *P* values were calculated using the two-tailed Student's *t*-test, *: $P < 0.05$; **: $P < 0.01$; ***: $P < 0.001$; ns: not statistically significant, $\alpha = 0.05$. The exact *P* values are provided in Source Data.

f, The frequency of CCP formation in non-transfected Cos7 cells and Cos7 cells transfected with siRNA for the control (luciferase), CIP4, FBP17, or Synd2 was quantified and is summarised. *P* values were calculated using the two-tailed Student's *t*-test, $\alpha = 0.05$. The exact *P* values are provided in Source Data. No significant differences existed between any two groups. Data are presented as mean ± standard deviation from four independent experiments. All data points are shown.

g, Additional examples of the correlative imaging of HS-AFM and confocal laser-scanning microscopy (CLSM). The fluorescent (FL) intensity of EGFP-fused CLTB and mCherry-fused CIP4 and the maximum membrane height at the CCP area were plotted against time. Time 0 was when the pit completely closed and is indicated with a black dotted line. Other details of the experimental conditions and data presentations are the same as those in Fig. 2d.

h, Additional examples of the time-lapse super-resolution SIM images obtained from Cos7 cells expressing EGFP-fused CLTB and mCherry-fused CIP4. Details of the experimental conditions and data presentations are the same as those in Fig. 2e.

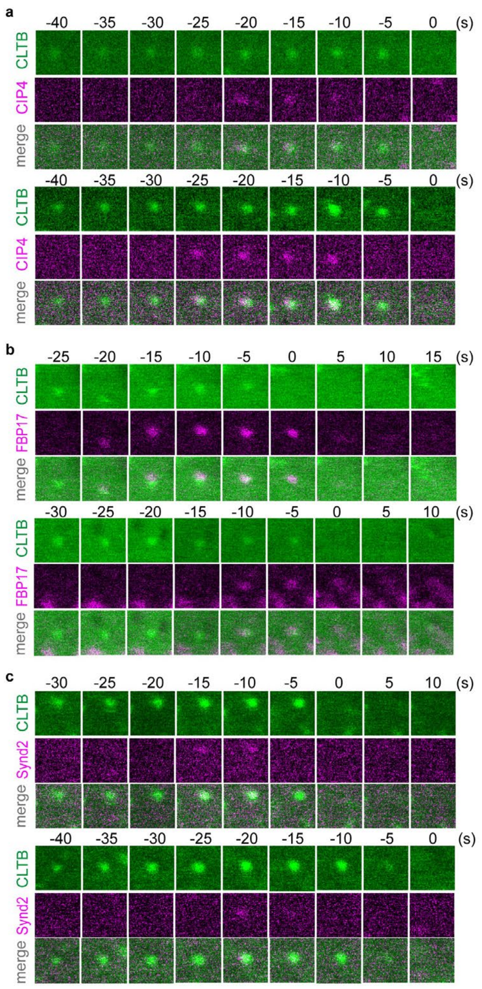

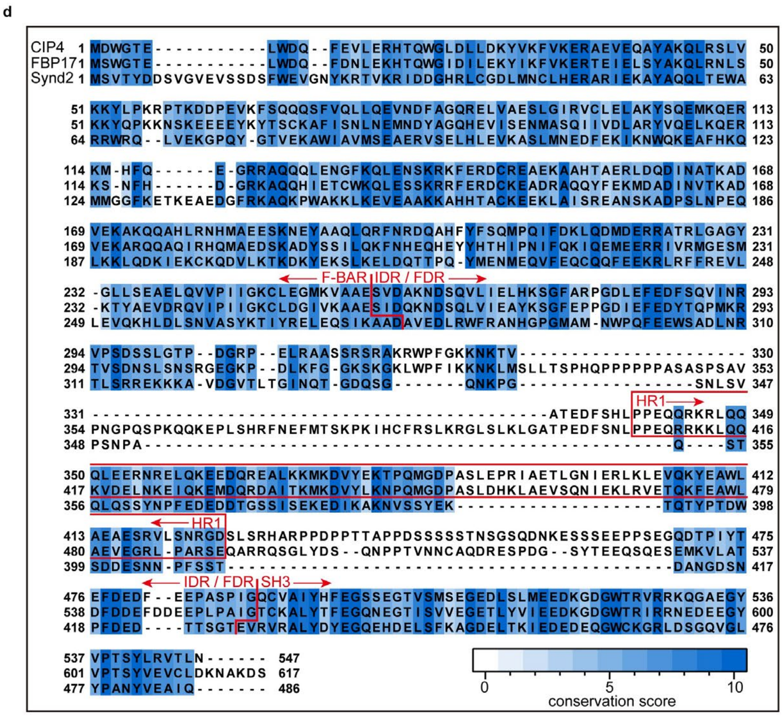

e

similarity (%) domain / domain	CIP4-F-BAR	similarity (%) domain / domain	CIP4-FDR	similarity (%) domain / domain	CIP4-SH3
FBP17-F-BAR	79.4	FBP17-FDR	52.6	FBP17-SH3	70.8
Synd2-F-BAR	39.3	Synd2-IDR	13.8	Synd2-SH3	46.7

Supplementary Figure. 3 Assembly of F-BAR proteins CIP4, FBP17, and Synd2 to CCP.

a–c, Additional examples of the time-lapse fluorescence images obtained from Cos7 cells expressing EGFP-fused CLTB and mCherry-fused CIP4 (a), FBP17 (b), or Synd2 (c). Details of the experimental conditions and data presentations are the same as those in Fig. 2a.

d, Amino acid sequence alignment of *Mus musculus* CIP4 (Uniprot, Q8CJ53-3), *Homo sapiens* FBP17 (Uniprot, Q96RU3), and *Mus musculus* Synd2 (Uniprot, Q9WVE8). Alignment was performed using T-Coffee. The conservation was scored and presented as indicated in a colour bar. The F-BAR, FDR/IDR, HR1, and SH3 domains are indicated.

e, Pairwise-domain similarity among CIP4, FBP17, and Synd2. The analysis was performed using the EMBOSS Needle.

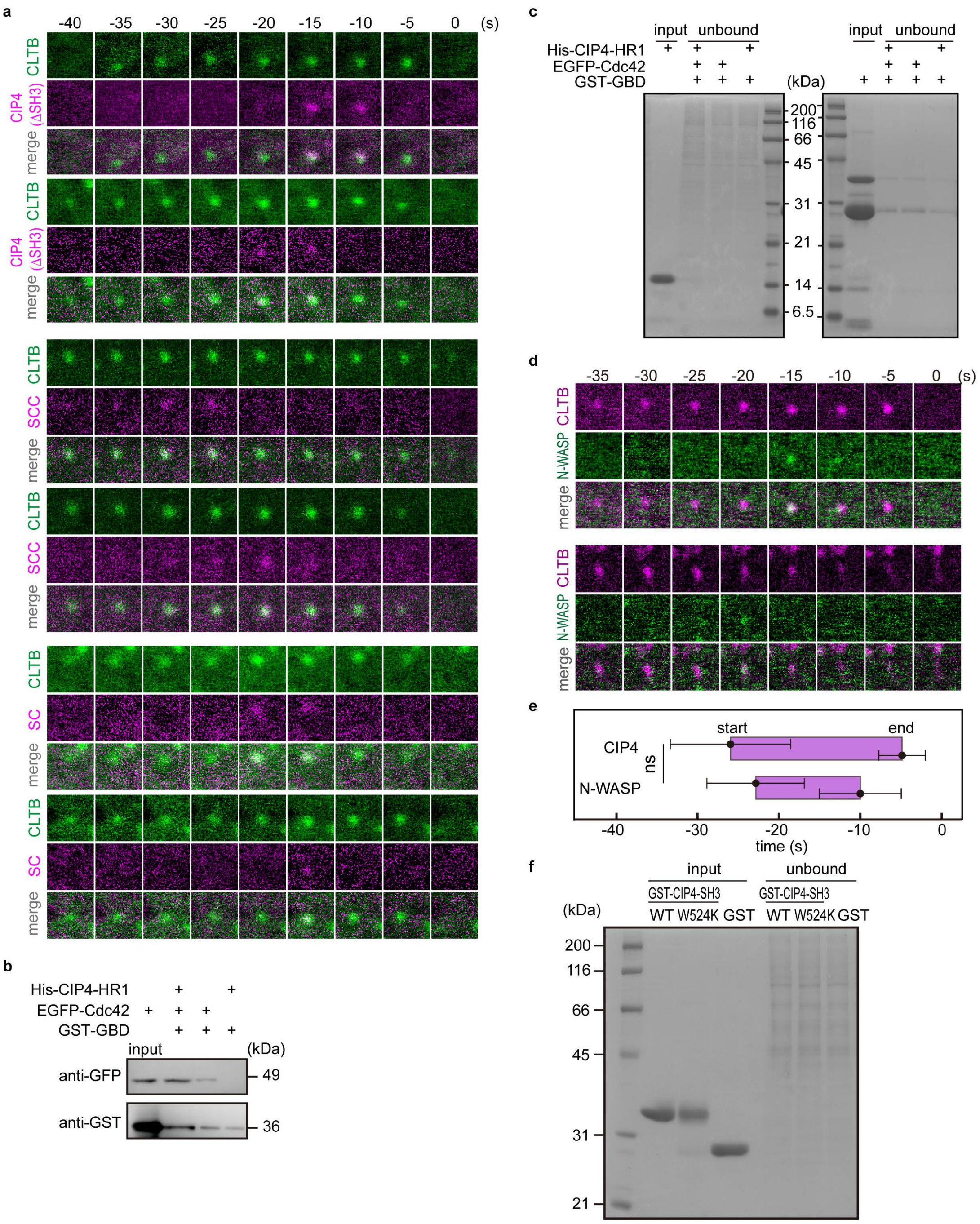
a
-40
-35
-30
-25
-20
-15
-10
-5
0
(s)
CLTB
CIP4 (ΔSH3)
merge
SCC
SC
b
His-CIP4-HR1
EGFP-Cdc42
GST-GBD
input
(kDa)
anti-GFP
49
anti-GST
36
c
input
unbound
(kDa)
200
116
66
45
31
21
14
6.5
d
N-WASP
e
start
end
CIP4
ns
N-WASP
time (s)
f
GST-CIP4-SH3
WT
W524K
GST

Supplementary Figure. 4 FDR and SH3 domain are necessary for asymmetric bulge formation.

a, Additional examples of the time-lapse fluorescence images obtained from Cos7 cells expressing EGFP-fused CLTB and mCherry-fused chimeric CIP4 molecules. Details of the experimental conditions and data presentations are the same as those in Fig. 3b.

b, c, Pull-down assay among CIP4-HR1, Cdc42, and the GBD of N-WASP. GST-tagged GBD was incubated with or without hexahistidine-tagged CIP4-HR1 domain and the cell lysate containing EGFP-fused Cdc42. The eluted fractions were subjected to western blot analysis using an anti-GFP antibody (b). The input amount of CIP4-HR1 and the unbound fraction were analysed using SDS-PAGE (c). The experiment was repeated twice and similar results were obtained.

d, Time-lapse fluorescence images of EGFP-fused N-WASP and mCherry-fused CLTB expressed in Cos7 cells. Time 0 was when the clathrin signal disappeared. The image size is 1.0 ×1.0 μm^2.

e, A summary of the assembly profile of CIP4 and N-WASP at CCPs. Time 0 was when the clathrin signal disappeared. The timing when the fluorescence signal appeared and disappeared at the CCP area was defined as 'start' and 'end', respectively, and are plotted as mean ± standard deviation (N = 20 pits examined over three independent experiments, for each condition). *P* values were calculated using the two-tailed Student's t-test, ns: not statistically significant, $\alpha = 0.05$. The exact *P* values are provided in Source Data.

f, Input amount of GST-tagged CIP4-SH3(WT), CIP4-SH3(W524K), empty GST, and the pull-down assay's unbound fraction in Fig. 3e was analysed using SDS-PAGE. The experiment was repeated three times and similar results were obtained.

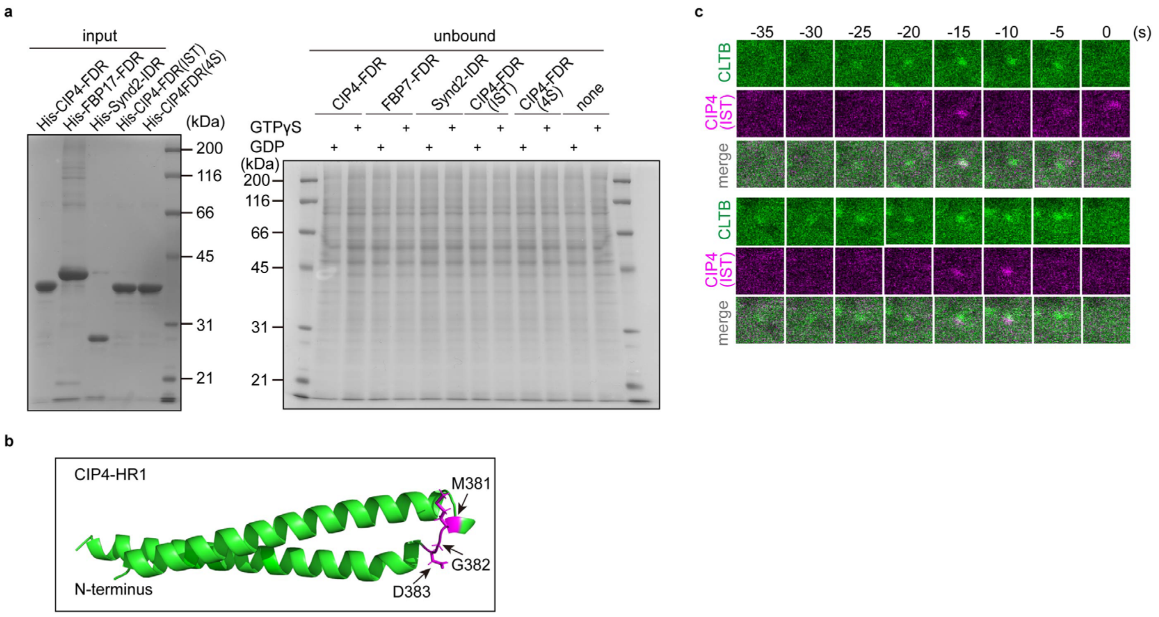

Supplementary Figure. 5 Cdc42 recruits CIP4 to the CCP site.

a, SDS-PAGE analysis of the input and the unbound fraction of the pull-down assay presented in Fig. 4a.

b, A three-dimensional structure of CIP4-HR1 predicted using AlphaFold2. Three mutated residues in CIP4(IST) (M381, G382, D383) are indicated in magenta.

c, Additional examples of the time-lapse fluorescence images of EGFP-fused CLTB and mCherry-fused CIP4(IST) expressed in Cos7 cells. Details of the experimental conditions and data presentations are the same as those in Fig. 4b.

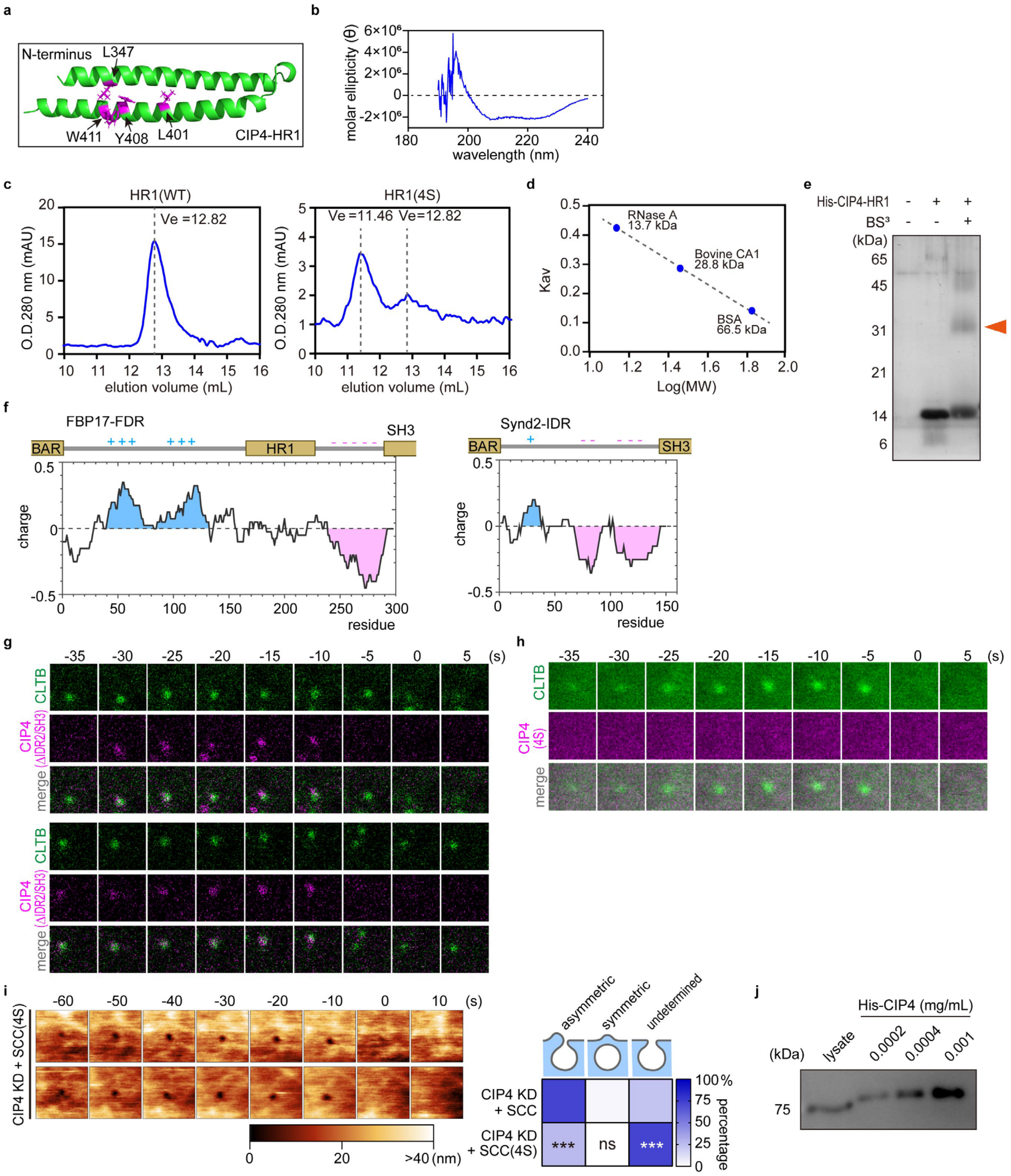
a
N-terminus
L347
W411
Y408
L401
CIP4-HR1
b
molar ellipticity (θ)
wavelength (nm)
c
HR1(WT)
Ve =12.82
HR1(4S)
Ve =11.46 Ve=12.82
O.D.280 nm (mAU)
elution volume (mL)
d
RNase A 13.7 kDa
Bovine CA1 28.8 kDa
BSA 66.5 kDa
Kav
Log(MW)
e
His-CIP4-HR1
BS³
(kDa)
f
FBP17-FDR
BAR
HR1
SH3
Synd2-IDR
charge
residue
g
CLTB
CIP4 (ΔIDR2/SH3)
merge
h
CLTB
CIP4 (4S)
merge
i
CIP4 KD + SCC(4S)
0
20
>40 (nm)
asymmetric
symmetric
undetermined
CIP4 KD + SCC
CIP4 KD + SCC(4S)
***
ns
***
percentage
j
His-CIP4 (mg/mL)
(kDa)
lysate
0.0002
0.0004
0.001
75

Supplementary Figure. 6 Structural and functional properties of FDRs.

a, Three-dimensional structure of the CIP4-HR1 domain predicted using AlphaFold2. The mutated residues in the CIP4-HR1(4S) are indicated in magenta.

b, Circular dichroism spectrum of purified CIP4-HR1.

c, Gel-filtration chromatography of CIP4-HR1(WT), and CIP4-HR1(4S). The absorbance at 280 nm (mAU) is plotted against the elution volume (mL). The peak position (V_e) is indicated with a dotted line.

d, A size calibration of gel-filtration chromatography shown in b. RNase A (13.7 kDa), bovine carbonic anhydrase 1 (bovine CA-1, 28.8 kDa), and bovine serum albumin (BSA, 66.5 kDa) were analysed. The partition coefficient (K_{av}) obtained from the chromatogram is plotted against the molecular weight.

e, Crosslinking assay of CIP4-HR1. Non-treated and bis(sulfosuccinimidyl)suberate (BS^3)-treated CIP4-HR1(WT) were subjected to SDS-PAGE, followed by silver staining. Band of speculated HR1 dimer is indicated with an arrowhead. The experiment was repeated three times and similar results were obtained.

f, Charge plot of Synd2-IDR and FBP17-FDR. Positively and negatively charged regions are marked in blue and red, respectively.

g, Additional examples of the time-lapse fluorescence images of EGFP-fused CLTB and mCherry-fused CIP4 (ΔIDR2/SH3) expressed in Cos7 cells. Details of the experimental conditions and data presentations are the same as those in Fig. 5f.

h, Time-lapse fluorescence images of EGFP-fused CLTB and mCherry-fused CIP4(4S) expressed in Cos7 cells. The image size was 1.0 ×1.0 μm^2. The time point of the disappearance of the clathrin signal was defined as '0 s'.

i, Time-lapse HS-AFM images obtained from Cos7 cells expressing EGFP-fused SCC(4S) under the CIP4-KD background. Images were taken every 10 s. Time 0 was when the pit completely closed on the HS-AFM image. The height information of the AFM image is presented using a colour bar. The image size was 1.0 × 1.0 μm^2. The frequency of three different closing patterns was measured and summarised in the right panel (N = 4 biologically independent cells, for each condition). *P* values were calculated by comparing the ratio of the same closing pattern between the non-treated and treated group using a two-tailed Student's t-test and

indicated in each block, ***: $P < 0.001$; ns: not statistically significant, $\alpha = 0.05$. The exact P values are provided in Source Data.

j, Endogenous amount of CIP4 in Cos7 cells. The amount of CIP4 in the total cell lysate of Cos7 cells was detected using western blotting and compared with *in vitro* purified Hexa-histidine-tagged CIP4 with known concentration. The experiment was repeated three times and similar results were obtained.

Insert	Vector/Template	Enzyme/Polymerase	Forward (5'-3')	Reverse (3'-5')
CIP4	pEGFP-C1	Xho I/Kpn I	AAACTCGAGACATGGATTGGGGTACCGAGT	AAAGGTACCTCAGTTGAGTGTGACTCGGAG
CIP4	pmCherry-C1	Xho I/Kpn I	AAACTCGAGACATGGATTGGGGTACCGAGT	AAAGGTACCTCAGTTGAGTGTGACTCGGAG
CIP4	pET-28a(+)	EcoR I/Sal I	AAAGAATTCATGGATTGGGGTACCGAGTTG	AAAGTCGACTCAGTTGAGTGTGACTCGGAG
CIP4(IST)	pmCherry-C1-CIP4	PrimeSTAR® Max DNA Polymerase	CCACAAATCAGCACCCCTGCCAGCTTAGAGCCC	GGCAGGGGTGCTGATTTGTGGTGTTTTCTCATA
CIP4(ΔSH3/IDR2)	pmCherry-C1	Xho I/EcoR I	AAACTCGAGACATGGATTGGGGTACCGAGT	AAGAATTCACTGTCCCCTCGGTTACTGAGG
CIP4(L347S)	pmCherry-C1-CIP4	PrimeSTAR® Max DNA Polymerase	AAGCGAAGTCAGCAACAGCTGGAAGAG	TTGCTGACTTCGCTTTCTCTGCTGCTC
CIP4(L401S)	pmCherry-C1-CIP4	PrimeSTAR® Max DNA Polymerase	GAGAGGAGTAAGTTGGAAGTGCAGAAG	CAACTTACTCCTCTCAATGTTGCCCAG
CIP4(Y408S)	pmCherry-C1-CIP4	PrimeSTAR® Max DNA Polymerase	CAGAAGAGTGAGGCTTGGTTGGCAGAA	AGCCTCACTCTTCTGCACTTCCAACTT
CIP4(W411S)	pmCherry-C1-CIP4	PrimeSTAR® Max DNA Polymerase	GAGGCTAGTTTGGCAGAAGCTGAAAGC	TGCCAAACTAGCCTCATACTTCTGCAC
FBP17	pEGFP-C1	EcoR I/Sal I	AAAGAATTCAATGAGCTGGGGCACCGAGCT	AAGTCGACCTAGGAATCTTTGGCATTTTTG
FBP17	pmCherry-C1	EcoR I/Sal I	AAAGAATTCAATGAGCTGGGGCACCGAGCT	AAGTCGACCTAGGAATCTTTGGCATTTTTG
FBP17-FDR	pET-28a(+)	EcoR I/Sal I	AAAGAATTCTCAATTGATCAGAAAAATGAT	AAAGTCGACCTATATGGCAGGGAGGGGCTC
Syndapin2	pEGFP-C1	Bgl II/Sac I	AAAGATCTCCGATGTCTGTCACCTACGATG	AAAGTCGACTCACTGGATAGCCTCGACATA
Syndapin2	pmCherry-C1	Bgl II/Sac I	AAAGATCTCCGATGTCTGTCACCTACGATG	AAAGTCGACTCACTGGATAGCCTCGACATA
Syndapin2-IDR	pET-28a(+)	EcoR I/Sal I	AAAGAATTCGCAGCAGATGCGGTAGAGGAC	AGTCGACCTATTCTGTTCCTGAGGTCGTGT
CIP4-ΔSH3	pmCherry-C1	Xho I/EcoR I	AAACTCGAGACATGGATTGGGGTACCGAGT	ACAAGAATTCTCAGCCGATAGGGGATGCAG
CIP4-F-BAR	pmCherry-C1	Xho I/EcoR I	AAACTCGAGACATGGATTGGGGTACCGAGT	AAGAATTCTCACTCGGCGGCCACCTTCATG
CIP4-FDR	pmCherry-C1	Bgl II/Hind III	AAAAGATCTTCTGTGGATGCTAAGAACGAC	ACCGAAGCTTTCAGCCGATAGGGGATGCAG
CIP4-FDR	pET-28a(+)	EcoR I/Sal I	AAAGAATTCGGCTGCTCTGTGGATGCTAAGAACGACTC	AAGTCGACTCAACACTGGCCGATAGGGGAT
CIP4-FDR(IST)	pET-28a(+)-CIP4-FDR	PrimeSTAR® Max DNA Polymerase	CCACAAATCAGCACCCCTGCCAGCTTAGAGCCC	GGCAGGGGTGCTGATTTGTGGTGTTTTCTCATA
CIP4-FDR(L347S)	pET-28a(+)-CIP4-FDR	PrimeSTAR® Max DNA Polymerase	AAGCGAAGTCAGCAACAGCTGGAAGAG	TTGCTGACTTCGCTTTCTCTGCTGCTC
CIP4-FDR(L401S)	pET-28a(+)-CIP4-FDR	PrimeSTAR® Max DNA Polymerase	GAGAGGAGTAAGTTGGAAGTGCAGAAG	CAACTTACTCCTCTCAATGTTGCCCAG
CIP4-FDR(Y408S)	pET-28a(+)-CIP4-FDR	PrimeSTAR® Max DNA Polymerase	CAGAAGAGTGAGGCTTGGTTGGCAGAA	AGCCTCACTCTTCTGCACTTCCAACTT
CIP4-FDR(W411S)	pET-28a(+)-CIP4-FDR	PrimeSTAR® Max DNA Polymerase	GAGGCTAGTTTGGCAGAAGCTGAAAGC	TGCCAAACTAGCCTCATACTTCTGCAC
CIP4-FDR(ΔHR1)	pET-28a(+)	EcoR I/Sal I	TCGCGGATCCGAATTCGGCTGCTCTGTGGATGCTA	CTAGCGTGACGGCTTAGGCTCAAGTGACTGAAATCTTCGG
			CCGAAGATTTCAGTCACTTGAGCCTAAGCCGTCACGCTAG	GCCGCAAGCTTGTCGACTCAGCCGATAGGGGATGCA
CIP4-FDR(ΔIDR1)	pET-28a(+)	EcoR I/Sal I	AAAGAATTCGGCTGCCCCCCGGAGCAGCAGAGAAA	AAAGTCGACTCAGCCGATAGGGGATGCAGG
CIP4-FDR(ΔIDR2)	pET-28a(+)	EcoR I/Sal I	AAGAATTCGGCTGCTCTGTGGATGCTAAGAACGAC	AAGAATTCACTGTCCCCTCGGTTACTGAGG
CIP4-SH3	pGEX-6P-1	EcoR I/Sal I	AAAGAATTCCAGTGTGTGGCTATCTACCAT	AAAGTCGACTCAGTTGAGTGTGACTCGGAG
CIP4-SH3(W524K)	pGEX-6P-1-CIP4-SH3	PrimeSTAR® Max DNA Polymerase	GATGGAAAGACGCGGGTCAGGAGGAAA	CCGCGTCTTTCCATCACCCTTGTCTTC
CIP4-HR1	pET-28a(+)	EcoR I/Sal I	AAAAGAATTCCCCCCGGAGCAGCAGAGAAA	AAGTCGACTCAGTCCCCTCGGTTACTGAGG
CIP4-HR1(L347S)	pET-28a(+)-CIP4-HR1	PrimeSTAR® Max DNA Polymerase	AAGCGAAGTCAGCAACAGCTGGAAGAG	TTGCTGACTTCGCTTTCTCTGCTGCTC
CIP4-HR1(L401S)	pET-28a(+)-CIP4-HR1	PrimeSTAR® Max DNA Polymerase	GAGAGGAGTAAGTTGGAAGTGCAGAAG	CAACTTACTCCTCTCAATGTTGCCCAG
CIP4-HR1(Y408S)	pET-28a(+)-CIP4-HR1	PrimeSTAR® Max DNA Polymerase	CAGAAGAGTGAGGCTTGGTTGGCAGAA	AGCCTCACTCTTCTGCACTTCCAACTT
CIP4-HR1(W411S)	pET-28a(+)-CIP4-HR1	PrimeSTAR® Max DNA Polymerase	GAGGCTAGTTTGGCAGAAGCTGAAAGC	TGCCAAACTAGCCTCATACTTCTGCAC
SCC	pEGFP-C1	Hind III II/Kpn I	AAAGCTTAAATGTCTGTCACCTACGATGAC	GGCCACCTTCATGCCCTCGAGCTCCCGGTAAATGGTTTTA
			TAAAACCATTTACCGGGAGCTCGAGGGCATGAAGGTGGCC	AAAGGTACCTCAGTTGAGTGTGACTCGGAG
SCC	pmCherry-C1	Hind III II/Kpn I	AAAGCTTAAATGTCTGTCACCTACGATGAC	AAAGGTACCTCAGTTGAGTGTGACTCGGAG
SCC(W524K)	pEGFP-C1-SCC	PrimeSTAR® Max DNA Polymerase	GATGGAAAGACGCGGGTCAGGAGGAAA	CCGCGTCTTTCCATCACCCTTGTCTTC
SCC(IST)	pEGFP-C1-SCC	PrimeSTAR® Max DNA Polymerase	CCACAAATCAGCACCCCTGCCAGCTTAGAGCCC	GGCAGGGGTGCTGATTTGTGGTGTTTTCTCATA
SCC(L347S)	pEGFP-C1-SCC	PrimeSTAR® Max DNA Polymerase	AAGCGAAGTCAGCAACAGCTGGAAGAG	TTGCTGACTTCGCTTTCTCTGCTGCTC
SCC(L401S)	pEGFP-C1-SCC	PrimeSTAR® Max DNA Polymerase	GAGAGGAGTAAGTTGGAAGTGCAGAAG	CAACTTACTCCTCTCAATGTTGCCCAG
SCC(Y408S)	pEGFP-C1-SCC	PrimeSTAR® Max DNA Polymerase	CAGAAGAGTGAGGCTTGGTTGGCAGAA	AGCCTCACTCTTCTGCACTTCCAACTT
SCC(W411S)	pEGFP-C1-SCC	PrimeSTAR® Max DNA Polymerase	GAGGCTAGTTTGGCAGAAGCTGAAAGC	TGCCAAACTAGCCTCATACTTCTGCAC
SC	pEGFP-C1	Hind III II/Kpn I	AAAGCTTAAATGTCTGTCACCTACGATGAC	AAGGTACCTCAGCCGATAGGGGATGCAGGC
SC	pmCherry-C1	Hind III II/Kpn I	AAAGCTTAAATGTCTGTCACCTACGATGAC	AAGGTACCTCAGCCGATAGGGGATGCAGGC
CS	pmCherry-C1	Hind III/Sal I	AAAAAGCTTCGATGGATTGGGGTACCGAGT	TGCTTTGATGCTCTGCTCGAGGCATTTGCCAATAATGGGA
			TCCCATTATTGGCAAATGCCTCGAGCAGAGCATCAAAGCA	AAAGTCGACTTCTGTTCCTGAGGTCGTGTC
Cdc42	pEGFP-C1	Xho I/EcoR I	AGCCTCGAGAAATGCAGACAATTAAGTGTG	AAAGAATTCAATCATAGCAGCACACACCTG
Cdc42	pET-28a(+)	EcoR I/Sal I	AAAGAATTCATGCAGACAATTAAGTGTGTT	AAAGTCGACTCATAGCAGCACACACCTGCG
Cdc42(Q61L)	pET-28a(+)-Cdc42	PrimeSTAR® Max DNA Polymerase	GCAGGGCTAGAGGATTATGACAGATTA	ATCCTCTAGCCCTGCAGTATCAAAAAG
N-WASP	pEGFP-C1	Xho I/EcoR I	AACTCGAGAAATGAGCTCCGTCCAGCAGCA	AGAATTCTCAGTCTTCCCACTCATCATCAT
N-WASP	pET-28a(+)	EcoR I/Hind III	AAAAGAATTCATGAGCTCCGTCCAGCAGCA	AAAAGCTTTCAGTCTTCCCACTCATCATCA
N-WASP-GBD	pGEX-6P-1	EcoR I/Xho I	AAGAATTCGATATAGGAACACCAAGCAATT	AGCGGCCGCTCAAATAAAGTCATATATAACT
CLTB	pEGFP-N1	Sac I/Kpn I	AAGAGCTCAAAATGGCTGATGACTTTGGCT	AAAGGTACCGAGCGGGACAGTGGCGTCTGC
CLTB	pmCherry-N1	Sac I/Kpn I	AAGAGCTCAAAATGGCTGATGACTTTGGCT	AAAGGTACCGAGCGGGACAGTGGCGTCTGC

Supplementary Table. 1 Primers used in the study.

A summary of the oligonucleotide primers used in this study. Primers were used to either amplify fragments of interest by PCR or generate point mutations by using PrimerSTAR® Max DNA Polymerase. Plasmids encoding SCC and CS chimeric molecules and CIP4-FDR(ΔHR1) were obtained by separately amplifying two different fragments, ligating, and subcloning into desired vectors (Methods), therefore, two pairs of primers were listed.
