# Supplementary material for: Self-assembly of CIP4 drives actin-mediated asymmetric pit-closing in clathrin-mediated endocytosis
Source: Nat Commun. 2023 Aug 1;14:4602. doi: 10.1038/s41467-023-40390-y (PMC10393992; doi:10.1038/s41467-023-40390-y)
Supplement: Supplementary file 3 — Description of Additional Supplementary Files [file 41467_2023_40390_MOESM3_ESM.pdf]

Description of Additional Supplementary Files

Supplementary Movie 1. Morphological changes of the plasma membrane during asymmetric CCP closing. Representative movie of asymmetric CCP closing in Cos7 cells captured using HS-AFM. Images were taken every 2 s. The height information of the AFM image is presented using a colour bar. Image size on the x-y plane: $1.0 \times 1.0\ \mu m^2$.

Supplementary Movie 2. Morphological changes of the plasma membrane during symmetric CCP closing. Representative movie of symmetric CCP closing in Cos7 cells captured using HS-AFM. Images were taken every 2 s. The height information of the AFM image is presented using a colour bar. Image size on the x-y plane: $1.0 \times 1.0\ \mu m^2$.

Supplementary Movie 3. Morphological changes of the plasma membrane during undetermined CCP closing. Representative movie of undetermined CCP closing in Cos7 cells captured using HS-AFM. Images were taken every 2 s. The height information of the AFM image is presented using a colour bar. Image size on the x-y plane: $1.0 \times 1.0\ \mu m^2$.
