# Supplementary material for: Self-assembly of CIP4 drives actin-mediated asymmetric pit-closing in clathrin-mediated endocytosis
Source: Nat Commun. 2023 Aug 1;14:4602. doi: 10.1038/s41467-023-40390-y (PMC10393992; doi:10.1038/s41467-023-40390-y)
Supplement: Supplementary file 7 — Reporting Summary [file 41467_2023_40390_MOESM7_ESM.pdf]

nature portfolio

Corresponding author(s): Shige H. Yoshimura

Last updated by author(s): Jul 17, 2023

Reporting Summary

Nature Portfolio wishes to improve the reproducibility of the work that we publish. This form provides structure for consistency and transparency in reporting. For further information on Nature Portfolio policies, see our Editorial Policies and the Editorial Policy Checklist.

Statistics

For all statistical analyses, confirm that the following items are present in the figure legend, table legend, main text, or Methods section.

n/a	Confirmed	
☐	☒	The exact sample size (*n*) for each experimental group/condition, given as a discrete number and unit of measurement
☐	☒	A statement on whether measurements were taken from distinct samples or whether the same sample was measured repeatedly
☐	☒	The statistical test(s) used AND whether they are one- or two-sided<br>*Only common tests should be described solely by name; describe more complex techniques in the Methods section.*
☒	☐	A description of all covariates tested
☒	☐	A description of any assumptions or corrections, such as tests of normality and adjustment for multiple comparisons
☐	☒	A full description of the statistical parameters including central tendency (e.g. means) or other basic estimates (e.g. regression coefficient) AND variation (e.g. standard deviation) or associated estimates of uncertainty (e.g. confidence intervals)
☐	☒	For null hypothesis testing, the test statistic (e.g. *F*, *t*, *r*) with confidence intervals, effect sizes, degrees of freedom and *P* value noted<br>*Give P values as exact values whenever suitable.*
☒	☐	For Bayesian analysis, information on the choice of priors and Markov chain Monte Carlo settings
☒	☐	For hierarchical and complex designs, identification of the appropriate level for tests and full reporting of outcomes
☒	☐	Estimates of effect sizes (e.g. Cohen's *d*, Pearson's *r*), indicating how they were calculated

Our web collection on statistics for biologists contains articles on many of the points above.

Software and code

Policy information about availability of computer code

Data collection	High-speed atomic force microscopy images and correlative images were obtained by a tip-scan type HS-AFM system (BIXAMTM; Olympus Corporation) combined with a confocal microscope (FV1200, Olympus Corporation) with accompanying software, AFM Scanning System Software 1.6.0.12 and FV10-ASW 4.2. Fluorescence images were obtained by a confocal laser scanning microscope (FV3000, Olympus Corporation) with accompanying software, FV-31S. Superresolution fluorescence images were obtained by a structured illumination microscopic system (Elyra 7 with Lattice SIM2; Zeiss) with accompanying software, Zeiss Enz blue edition 3.6.
Data analysis	ImageJ 1.52a, GIMP 2.10.18, Gwyddion 2.55, OriginPro 9.8.0.200.

For manuscripts utilizing custom algorithms or software that are central to the research but not yet described in published literature, software must be made available to editors and reviewers. We strongly encourage code deposition in a community repository (e.g. GitHub). See the Nature Portfolio guidelines for submitting code & software for further information.

Data

Policy information about availability of data

All manuscripts must include a data availability statement. This statement should provide the following information, where applicable:

- Accession codes, unique identifiers, or web links for publicly available datasets
- A description of any restrictions on data availability
- For clinical datasets or third party data, please ensure that the statement adheres to our policy

Amino-acid sequences of Homo sapiens CLTB, Mus musculus CIP4, Homo sapiens FBP17, Mus musculus Synd2, Homo sapiens Cdc42, and Homo sapiens N-WASP

can be obtained from UniProt database (accession number P09497 for CLTB, Q8CJ53-3 for CIP4, Q96RU3 for FBP17, Q9WVE8 for Synd2, P60953 for Cdc42, and O00401 for N-WASP). All data supporting the conclusions and findings included in this study are available within the article or Supplementary Information. Source data are provided with this paper. Any additional information required to reanalyse the data reported in this study is available from the corresponding author, [S.H.Y], upon reasonable request.

Research involving human participants, their data, or biological material

Policy information about studies with human participants or human data. See also policy information about sex, gender (identity/presentation), and sexual orientation and race, ethnicity and racism.

Reporting on sex and gender	*Use the terms sex (biological attribute) and gender (shaped by social and cultural circumstances) carefully in order to avoid confusing both terms. Indicate if findings apply to only one sex or gender; describe whether sex and gender were considered in study design; whether sex and/or gender was determined based on self-reporting or assigned and methods used. Provide in the source data disaggregated sex and gender data, where this information has been collected, and if consent has been obtained for sharing of individual-level data; provide overall numbers in this Reporting Summary. Please state if this information has not been collected. Report sex- and gender-based analyses where performed, justify reasons for lack of sex- and gender-based analysis.*
Reporting on race, ethnicity, or other socially relevant groupings	*Please specify the socially constructed or socially relevant categorization variable(s) used in your manuscript and explain why they were used. Please note that such variables should not be used as proxies for other socially constructed/relevant variables (for example, race or ethnicity should not be used as a proxy for socioeconomic status). Provide clear definitions of the relevant terms used, how they were provided (by the participants/respondents, the researchers, or third parties), and the method(s) used to classify people into the different categories (e.g. self-report, census or administrative data, social media data, etc.) Please provide details about how you controlled for confounding variables in your analyses.*
Population characteristics	*Describe the covariate-relevant population characteristics of the human research participants (e.g. age, genotypic information, past and current diagnosis and treatment categories). If you filled out the behavioural & social sciences study design questions and have nothing to add here, write "See above."*
Recruitment	*Describe how participants were recruited. Outline any potential self-selection bias or other biases that may be present and how these are likely to impact results.*
Ethics oversight	*Identify the organization(s) that approved the study protocol.*

Note that full information on the approval of the study protocol must also be provided in the manuscript.

Field-specific reporting

Please select the one below that is the best fit for your research. If you are not sure, read the appropriate sections before making your selection.

- [x] Life sciences
- [ ] Behavioural & social sciences
- [ ] Ecological, evolutionary & environmental sciences

For a reference copy of the document with all sections, see nature.com/documents/nr-reporting-summary-flat.pdf

Life sciences study design

All studies must disclose on these points even when the disclosure is negative.

Sample size	The sample size is described in the figure legends. No statistical methods were used to predetermine sample sizes.
Data exclusions	When the HS-AFM scanning is not stable, images were not collected.
Replication	Repetitive experiments were performed and successful.
Randomization	Cells were randomly selected for HS-AFM imaging and the scanning area was randomly picked at the cell surface. Samples of clathrin-coated pits were randomly collected for analysis. For other observations by fluorescence microscope, cells expressing the protein of interest were randomly selected.
Blinding	Experiments performed in this article were not blinded and this is consistent with what is published in the field.

Reporting for specific materials, systems and methods

We require information from authors about some types of materials, experimental systems and methods used in many studies. Here, indicate whether each material, system or method listed is relevant to your study. If you are not sure if a list item applies to your research, read the appropriate section before selecting a response.

Materials & experimental systems

n/a	Involved in the study	
☐	☒	Antibodies
☐	☒	Eukaryotic cell lines
☒	☐	Palaeontology and archaeology
☒	☐	Animals and other organisms
☒	☐	Clinical data
☒	☐	Dual use research of concern
☒	☐	Plants

Methods

n/a	Involved in the study	
☒	☐	ChIP-seq
☒	☐	Flow cytometry
☒	☐	MRI-based neuroimaging

Antibodies

Antibodies used

rabbit anti-CIP4 (A301-186A-T; Bethyl Laboratories Inc.), rabbit anti-Pacsin2 (NBP2-24481SS; Novus Biologicals LLC), rabbit anti-FNBP1 (NBP2-24526SS; Novus Biologicals LLC), rabbit anti-N-WASP (4848; Cell Signaling Technology), rabbit anti-GFP (598; Medical & Biological Laboratories Co., Ltd.), and mouse anti-β-actin (A5441; Sigma-Aldrich), goat anti-rabbit IgG (H+L) antibody (A-11036; Thermo Fisher Scientific), Goat anti-mouse IgG (H+L) antibody (NA931V; Cytiva).

Validation

The specificity of anti-CIP4, anti-Pacsin2, anti-FNBP1, and anti-N-WASP in western blot was confirmed by using the cell lysate from Cos7 cells expressing EGFP-fused CIP4, Pacsin2, FNBP1, or N-WASP. These antibodies recognised both endogenous and exogenous proteins or desire. The specificity of anti-GFP antibody in western blot was confirmed by using the cell lysate from Cos7 cells expressing EGFP.

Eukaryotic cell lines

Policy information about cell lines and Sex and Gender in Research

Cell line source(s)

Cos7 cells were purchased from Cell Engineering Division in RIKEN BioResource Center (BRC).

Authentication

Cos7 cells were authenticated by Cell Engineering Division in RIKEN BioResource Center (BRC).

Mycoplasma contamination

The Cos7 cell line was tested negative for mycoplasma contamination.

Commonly misidentified lines
(See ICLAC register)

No commonly misidentified lines were used in this study.
