# Supplementary material for: Self-assembly of CIP4 drives actin-mediated asymmetric pit-closing in clathrin-mediated endocytosis
Source: Nat Commun. 2023 Aug 1;14:4602. doi: 10.1038/s41467-023-40390-y (PMC10393992; doi:10.1038/s41467-023-40390-y)

Original Blot of Figure. 3e

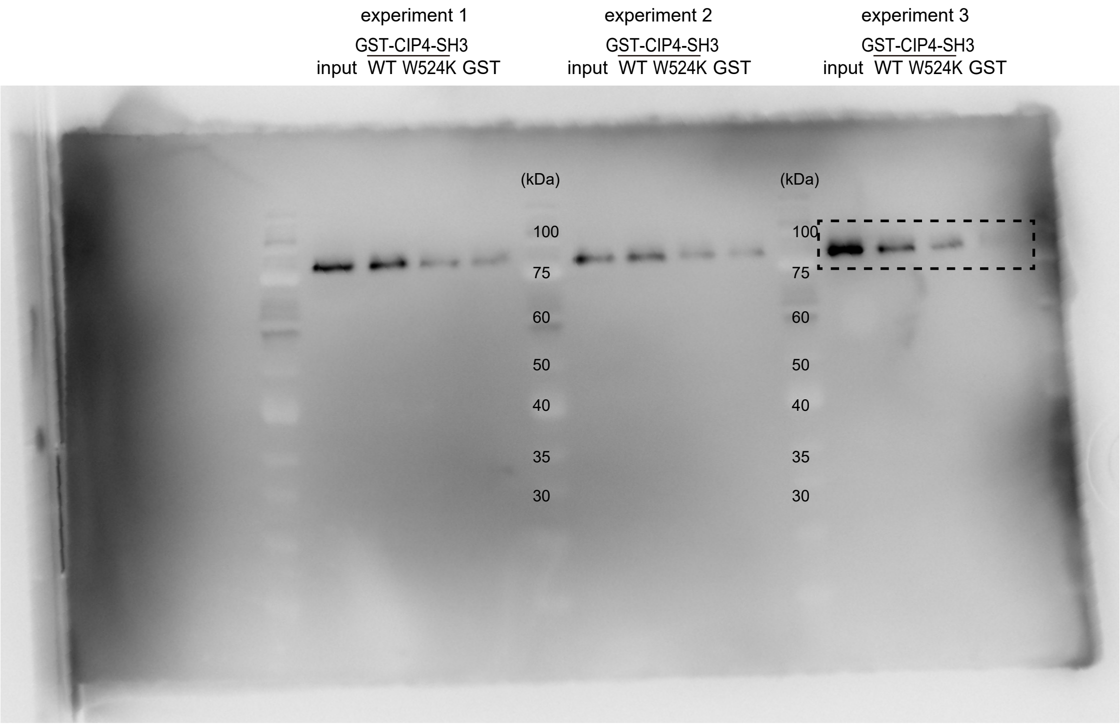

Original Blot and CBB staining of Figure. 4a

experiment 1 bound fraction

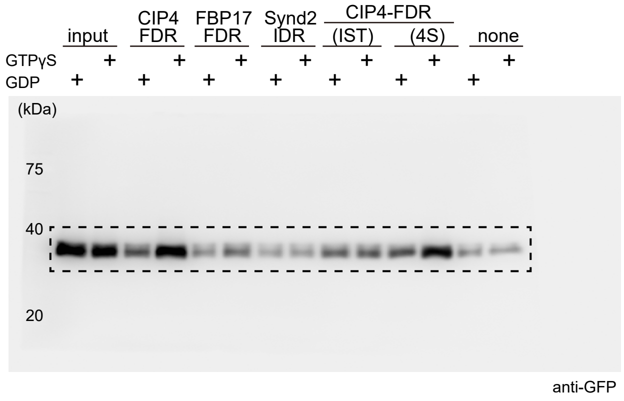

experiment 1 unbound fraction

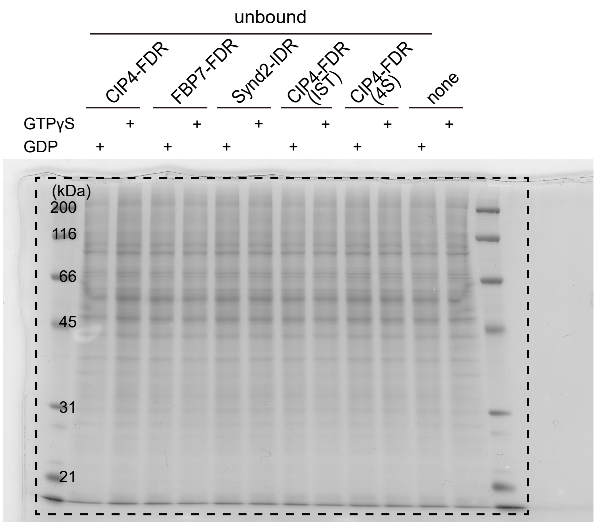

experiment 2 bound fraction

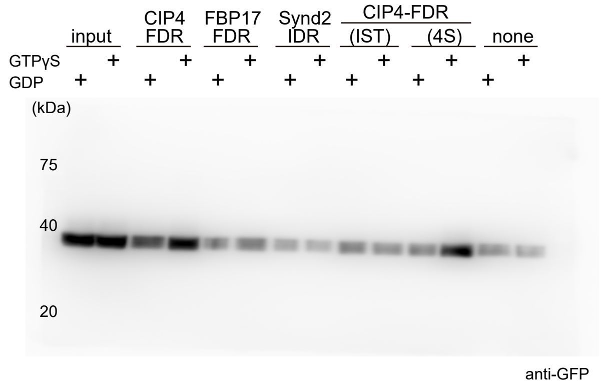

experiment 2 unbound fraction

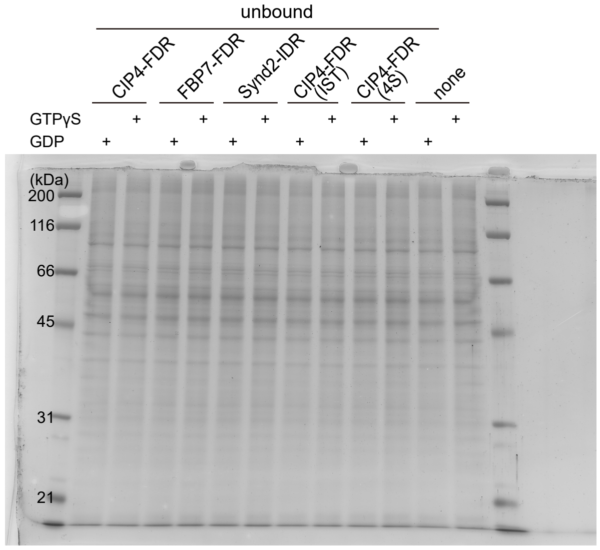

experiment 3 bound fraction

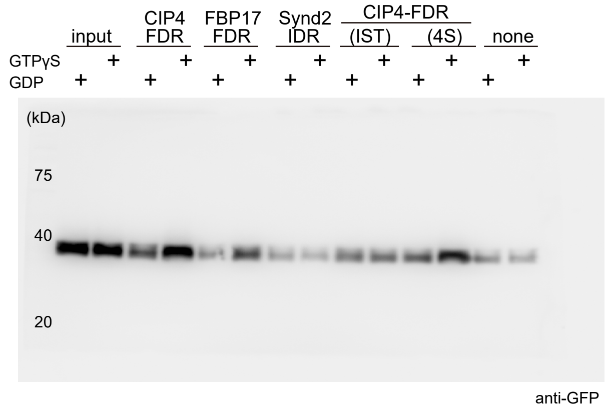

experiment 3 unbound fraction

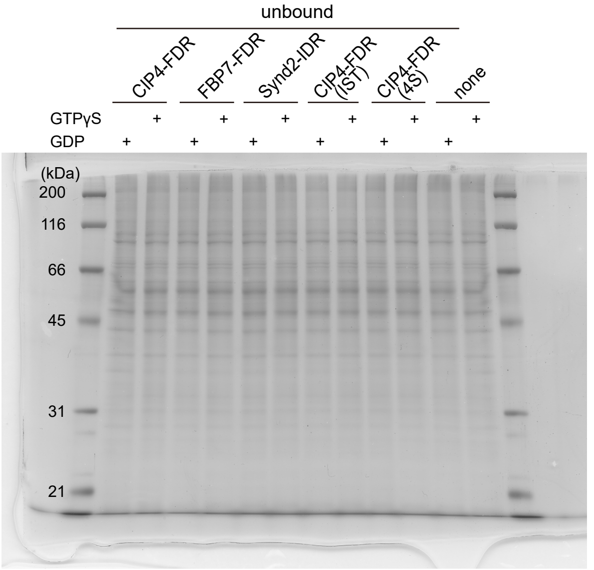

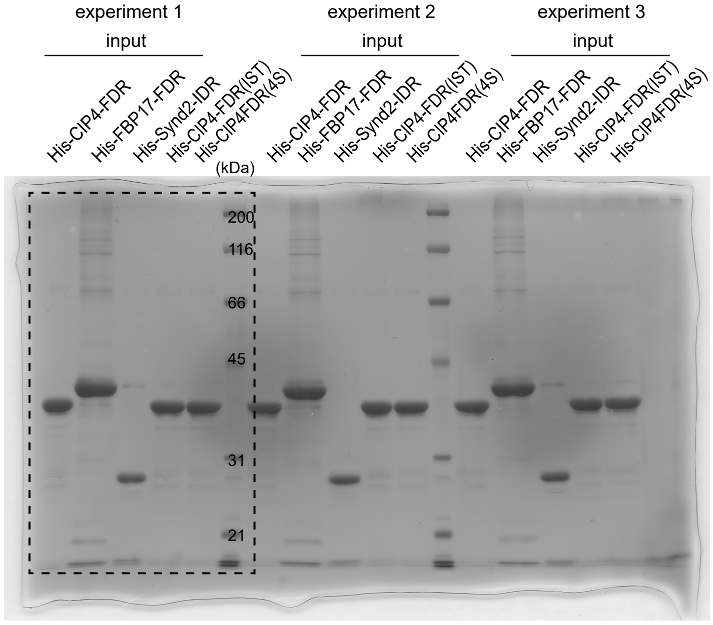

Original Blot of Supplementary Figure. 1e

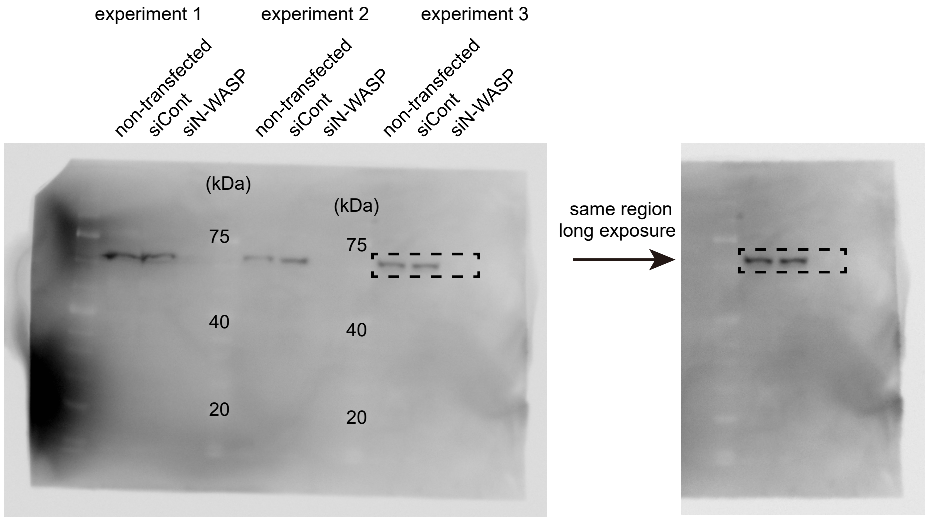

anti-N-WASP

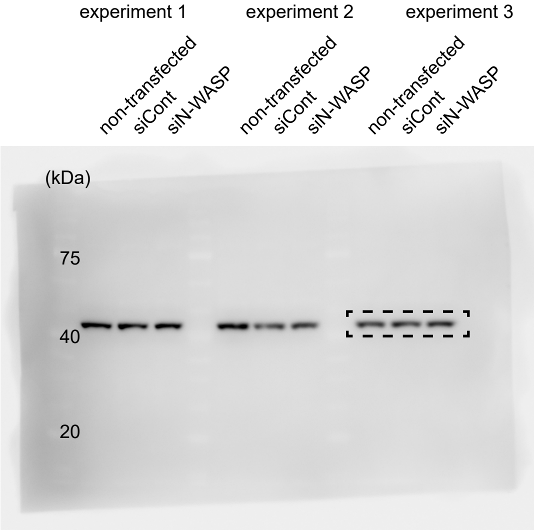

anti-β-actin

Original Blot of Supplementary Figure. 2a

experiment 1

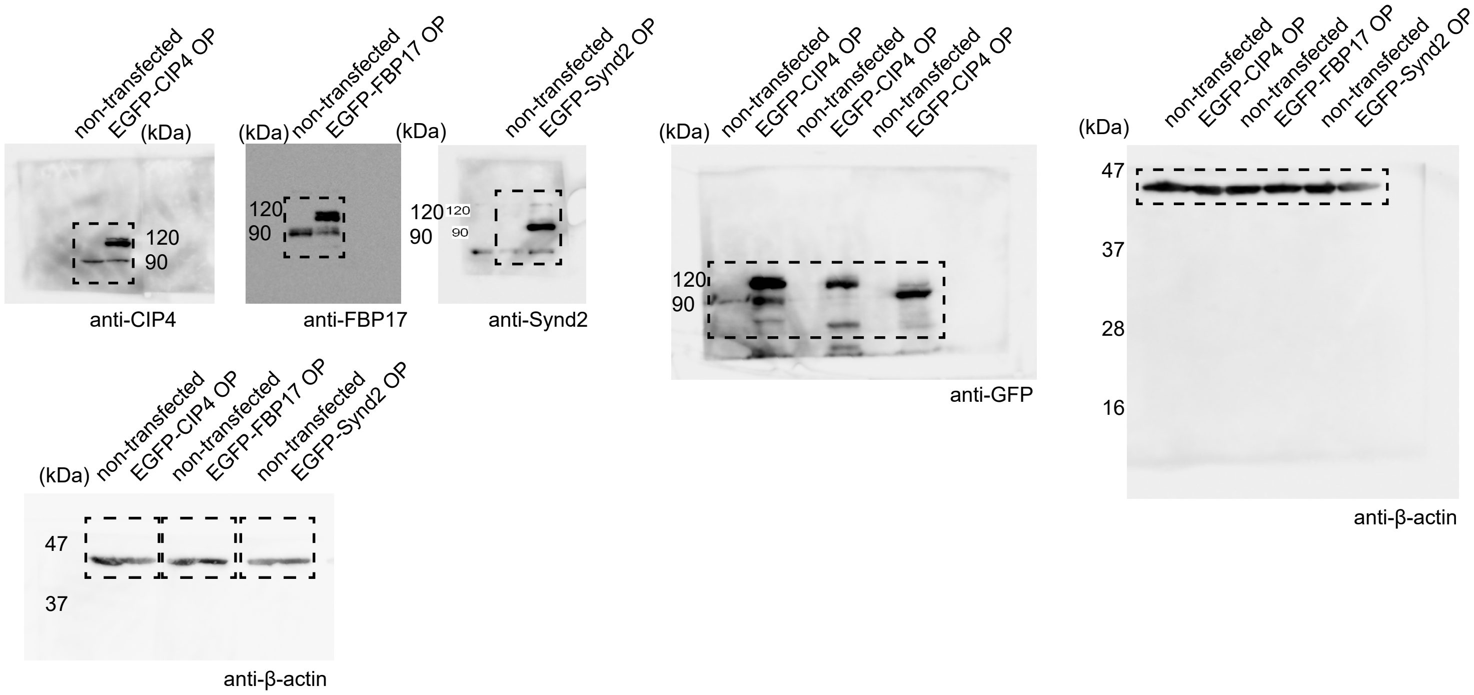

experiment 2

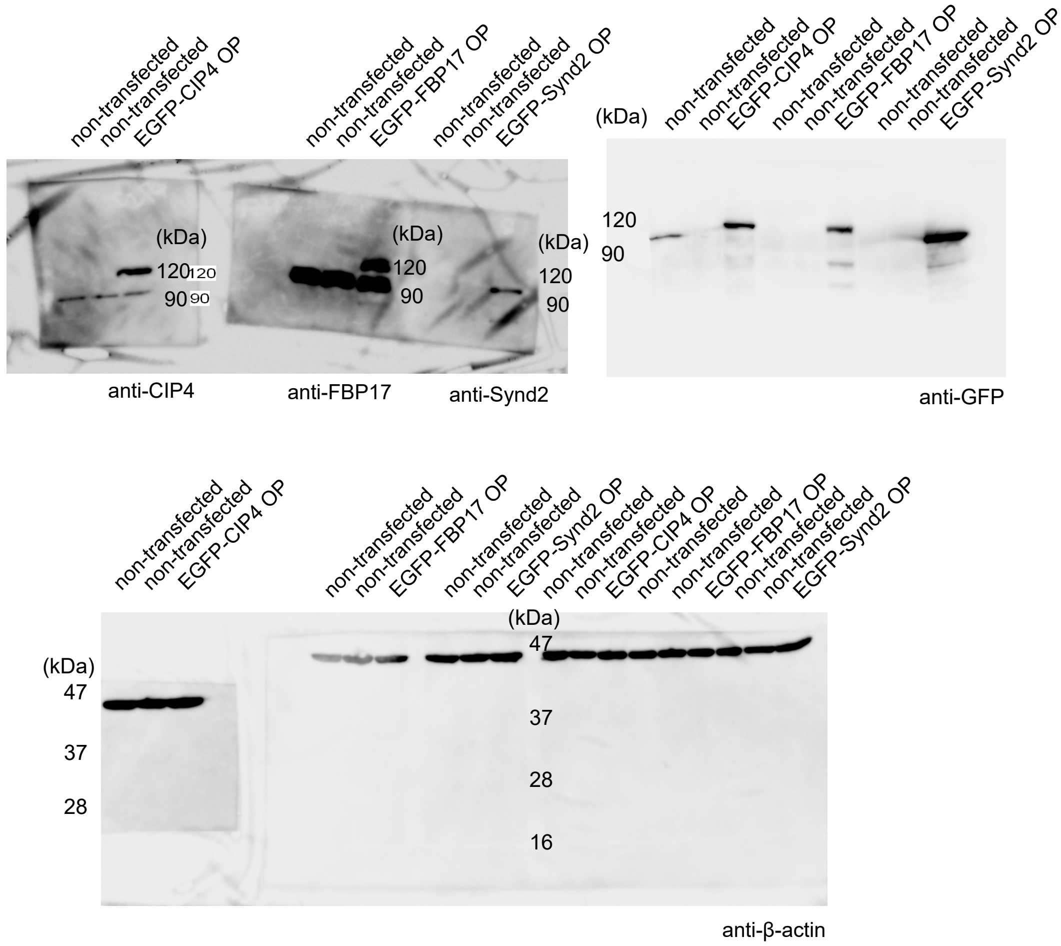

Original Blot of Supplementary Figure. 2a

experiment 3

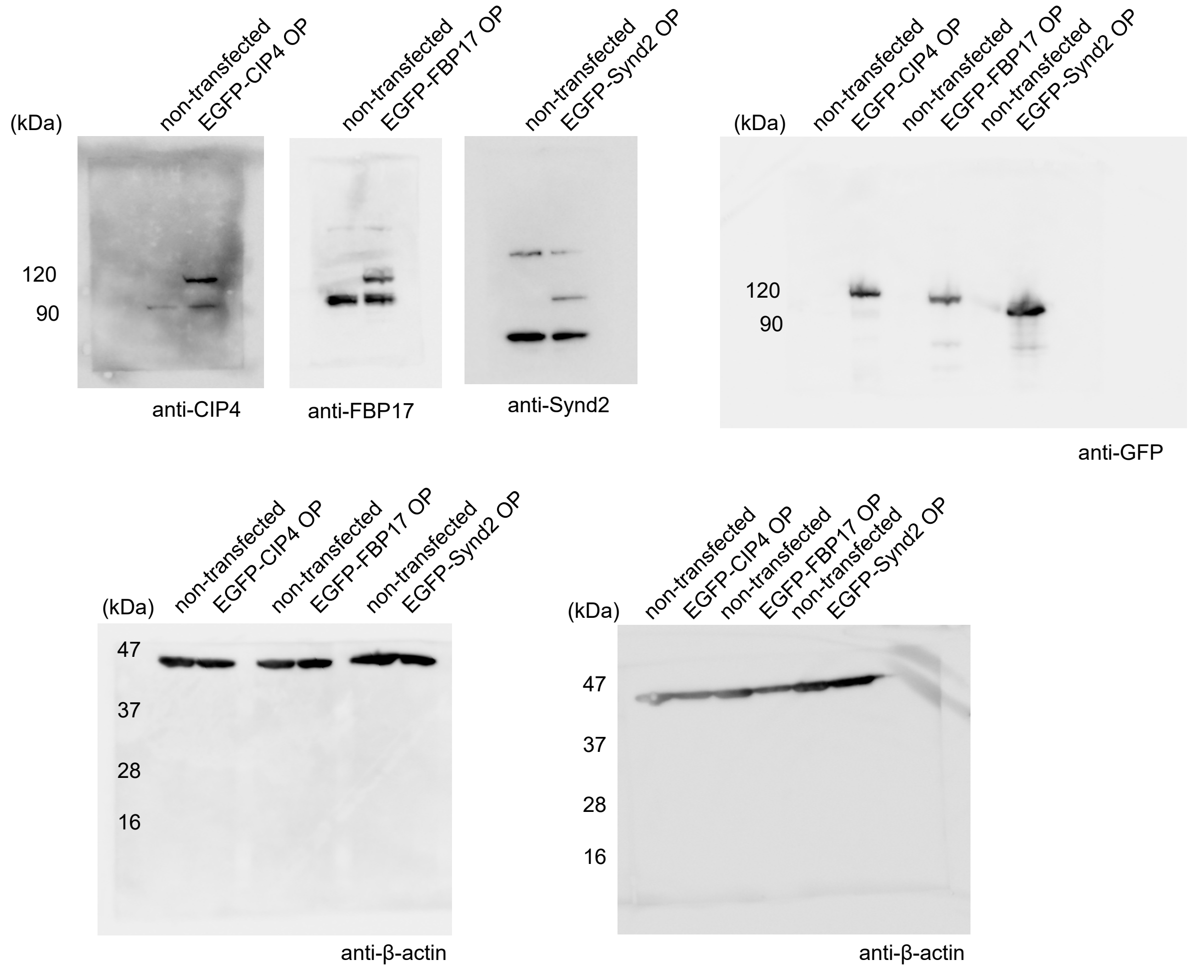

Original Blot of Supplementary Figure. 2c

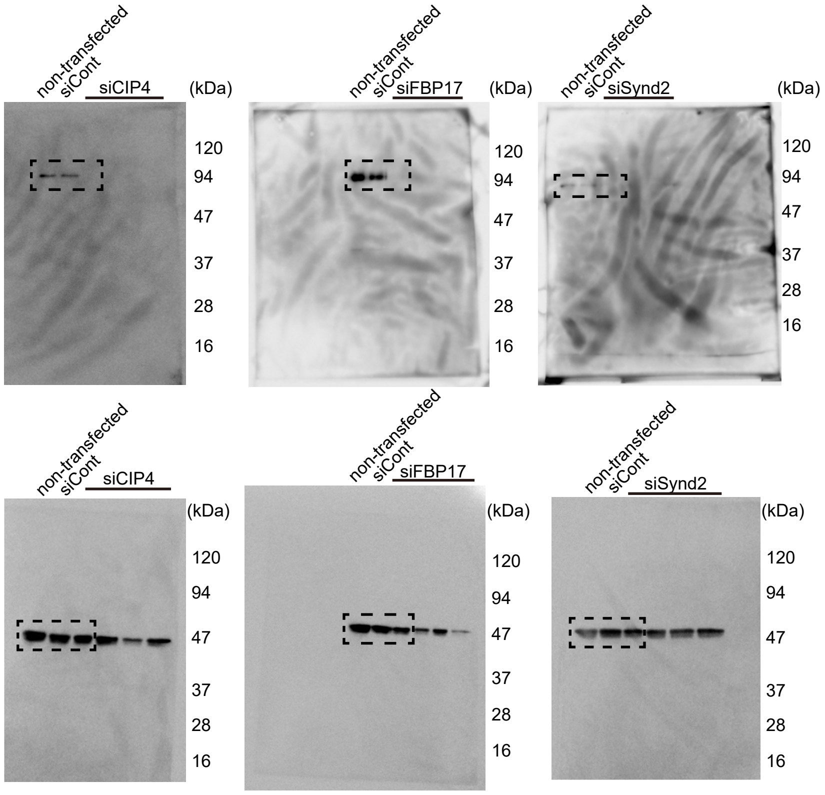

Original Blot of Supplementary Figure. 2e

experiment 1 experiment 2 experiment 3

EGFP-CIP4 + + + + + +
siCIP4_3' UTR + + + + + +
(kDa)

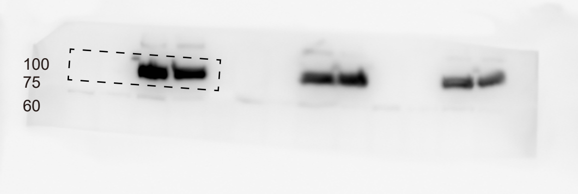

same gel, long exposure time

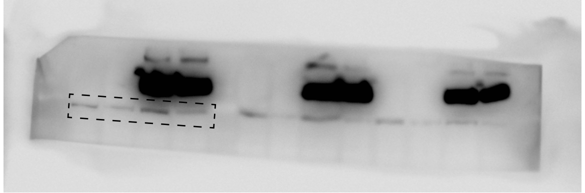

anti-CIP4

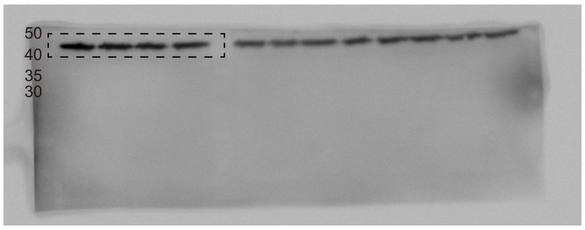

anti-β-actin

Original Blot of Supplementary Figure. 4b

experiment 1 experiment 2

His-Cip4-HR1 + + + +
EGFP-Cdc42 + + + +
GST-GBD + + + + + +
input

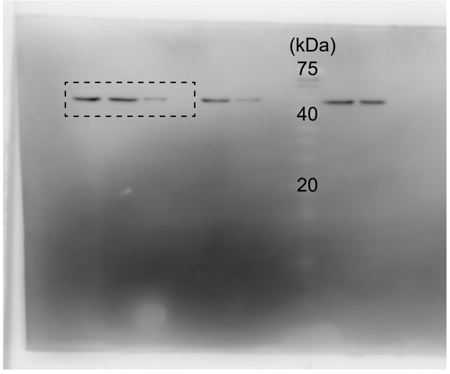

anti-GFP

experiment 1 experiment 2

His-Cip4-HR1 + + + +
EGFP-Cdc42 + + + +
GST-GBD input + + + + + +

(kDa)
75
40
20

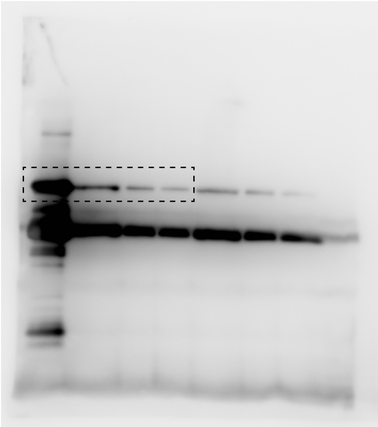

anti-GST

Original CBB staining of Supplementary Figure. 4c

experiment 1 experiment 2 experiment 1 experiment 2

His-Cip4-HR1 + + + + + + + +
EGFP-Cdc42 + + + + + + + +
GST-GBD + + + + + + + + + + + +
input input input input

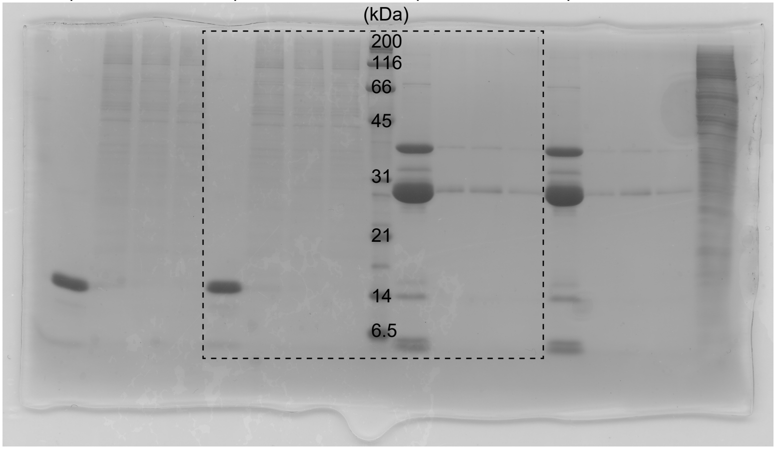

Original CBB staining of Supplementary Figure. 4f

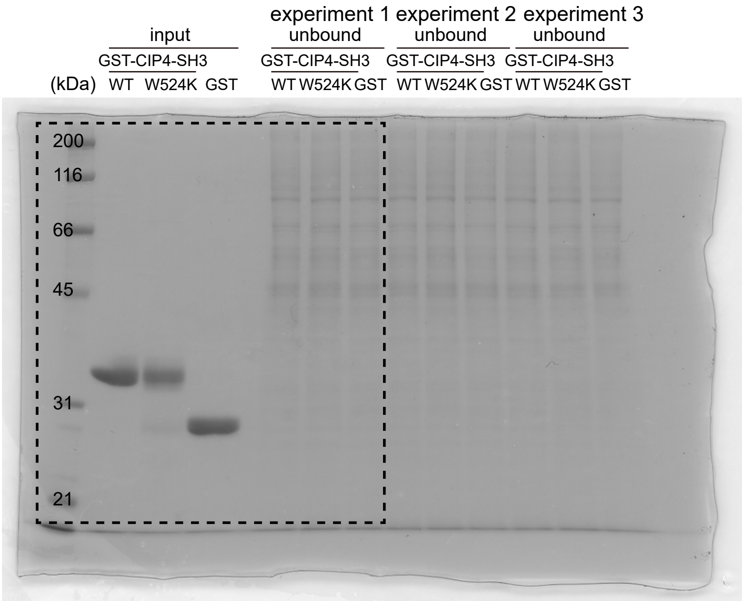

Original CBB staining of Supplementary Figure. 5a

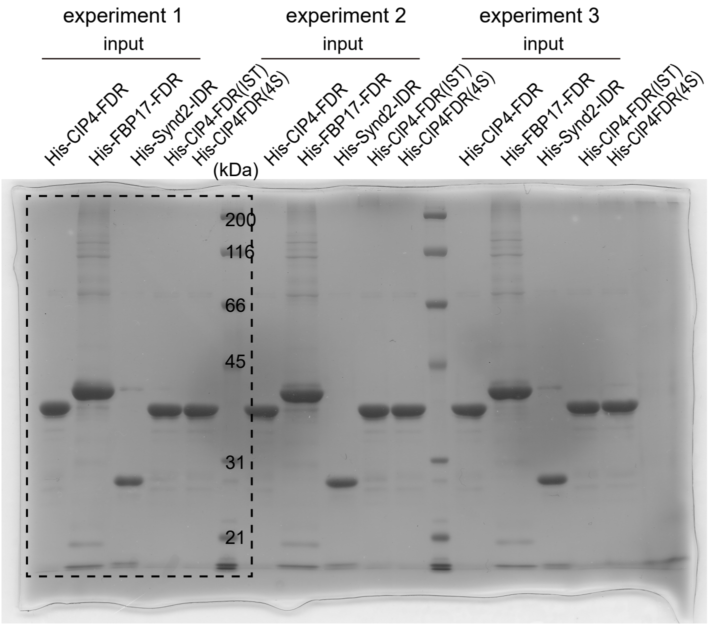

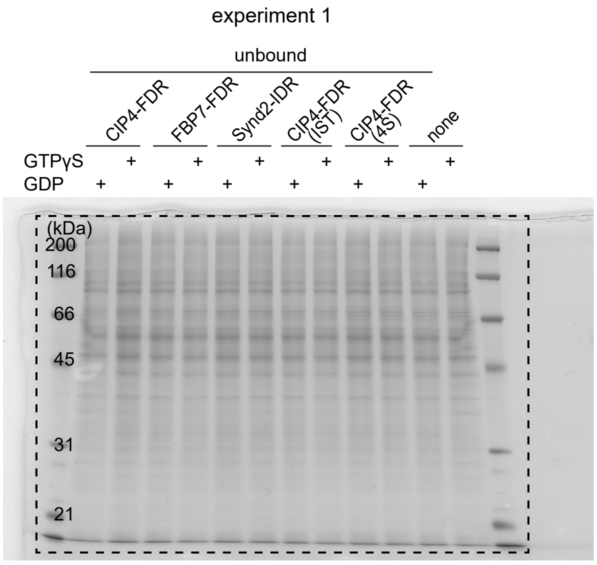

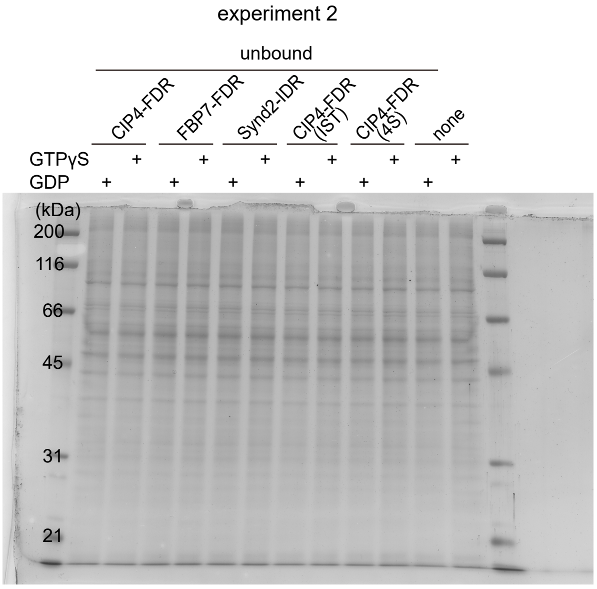

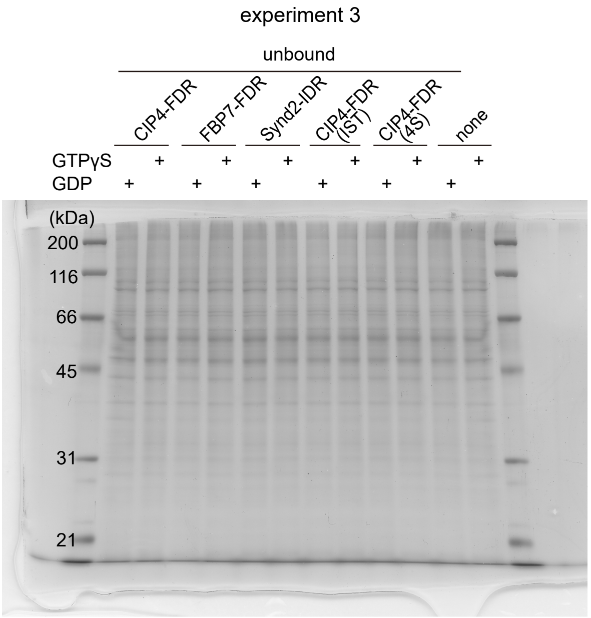

Original silver staining of Supplementary Figure. 6e

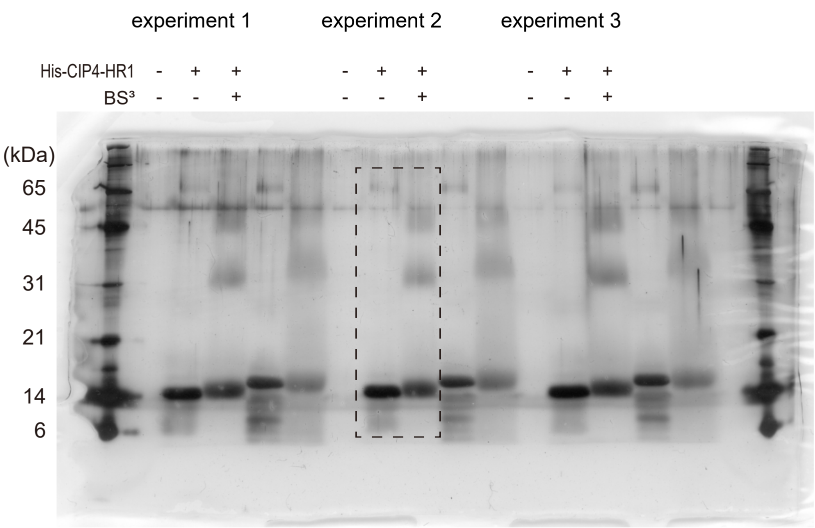

Original Blot of Supplementary Figure. 6j

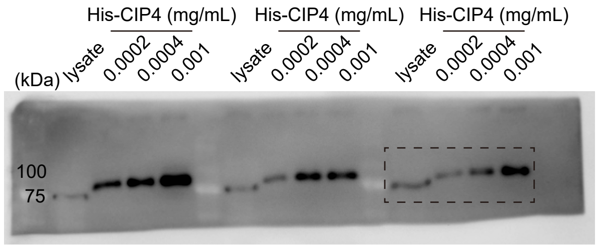

Supplement: Supplementary file 8 — Source Data [file 41467_2023_40390_MOESM8_ESM.zip › Yu and Yoshimura Original Blot.pdf]
